# White matter hyperintensity load is associated with premature brain aging

**DOI:** 10.18632/aging.204397

**Published:** 2022-11-30

**Authors:** Natalie Busby, Sarah Newman-Norlund, Sara Sayers, Roger Newman-Norlund, Sarah Wilson, Samaneh Nemati, Chris Rorden, Janina Wilmskoetter, Nicholas Riccardi, Rebecca Roth, Julius Fridriksson, Leonardo Bonilha

**Affiliations:** 1 Department of Communication Sciences and Disorders, University of South Carolina, Columbia, SC 29201, USA; 2 Department of Psychology, University of South Carolina, Columbia, SC 29201, USA; 3 Department of Health and Rehabilitation Sciences, Medical University of South Carolina, Charleston, SC 29425, USA; 4 Department of Neurology, Emory University, Atlanta, GA 30322, USA

**Keywords:** brain age, white matter hyperintensity, brain health, aging, health

## Abstract

Background: Brain age is an MRI-derived estimate of brain tissue loss that has a similar pattern to aging-related atrophy. White matter hyperintensities (WMHs) are neuroimaging markers of small vessel disease and may represent subtle signs of brain compromise. We tested the hypothesis that WMHs are independently associated with premature brain age in an original aging cohort.

Methods: Brain age was calculated using machine-learning on whole-brain tissue estimates from T1-weighted images using the BrainAgeR analysis pipeline in 166 healthy adult participants. WMHs were manually delineated on FLAIR images. WMH load was defined as the cumulative volume of WMHs. A positive difference between estimated brain age and chronological age (BrainGAP) was used as a measure of premature brain aging. Then, partial Pearson correlations between BrainGAP and volume of WMHs were calculated (accounting for chronological age).

Results: Brain and chronological age were strongly correlated (*r*(163)=0.932, *p*<0.001). There was significant negative correlation between BrainGAP scores and chronological age (*r*(163)=-0.244, *p*<0.001) indicating that younger participants had higher BrainGAP (premature brain aging). Chronological age also showed a positive correlation with WMH load (*r*(163)=0.506, *p*<0.001) indicating older participants had increased WMH load. Controlling for chronological age, there was a statistically significant relationship between premature brain aging and WMHs load (*r*(163)=0.216, *p*=0.003). Each additional year in brain age beyond chronological age corresponded to an additional 1.1mm^3^ in WMH load.

Conclusions: WMHs are an independent factor associated with premature brain aging. This finding underscores the impact of white matter disease on global brain integrity and progressive age-like brain atrophy.

## INTRODUCTION

Brain tissue atrophy is a pervasive phenomenon associated with aging [1, 2]. Nonetheless, recent studies modeling brain integrity with machine learning have indicated that brain aging is a process that can be decoupled from chronological age, where the brain can “age slower or faster” than would be expected based on chronological age [3, 4]. The reasons underlying the decoupling of chronological and brain aging remain unclear, but they may relate to the unique physiological and dynamic properties of the central nervous system, with brain plasticity serving as a protective mechanism [5]. Conversely, premature brain aging may reflect a unique susceptibility to chronic injury given to the brain’s high metabolic demand, particularly in the case of chronic insufficient cerebrovascular supply. To date, researchers have identified strong associations between age and systemic structural changes to the brain, including both changes in white matter, gray matter, and cerebrospinal fluid [6, 7].

To elucidate the mechanisms related to premature brain aging, it is important to consider white matter hyperintensities (WMHs). WMHs are neuroimaging markers of small vessel disease and are indicative of chronic insufficient cerebrovascular supply [8]. They have been previously regarded as benign findings but may represent signs of brain structural changes with a subtle relationship with cognitive performance [9, 10] in particular, executive functioning [11–14]. Even in younger individuals with low WMH load, a relationship between WMH load and working memory has been reported [11]. The association between chronological age and WMH load is well-reported, where older individuals have a higher WMH load [11]. Due to this relationship, recent research has also sought to predict chronological age using measures of WMH load [15]. However, these studies typically focus on the relationship between WMH load and *chronological* age, rather than brain age. More specifically, an association between quantifiable load of WMHs and premature brain aging would imply a relationship between small vessel disease and premature brain aging either as a direct link or through a shared common cause. It is important to note that estimates of cortical integrity using measures of brain age typically use structural T1-weighted MRI scans, whilst other modalities are more appropriate for the quantification of age-related WMH load (i.e., T2-weighted or T2-FLAIR) [16, 17]. The necessity of these different modalities is one reason why research has typically focused on the relationship between WMH load and chronological age rather than brain age. Despite this, the relationship between WMH severity and age-related changes have been previously investigated, for example, Habes and colleagues found that individuals with higher WMH burden also had age-related brain atrophy [18], however, to our knowledge no one has previously explored the relationship between WMH and brain age using the well-known software package BrainAgeR [19], which is capable of estimating differences between chronological age and brain-metric based age [3] and has been shown to out-perform other methods of estimating brain age [20]. Therefore, we tested this relationship and evaluated the independent association between quantifiable WMHs and brain age using the BrainAgeR analysis pipeline. We hypothesized that a higher WMH load is linearly associated with premature brain aging controlling for chronological age.

## MATERIALS AND METHODS

### Participants

Local healthy adult participants (N=166) were part of the Aging Brain Cohort at the University of South Carolina (ABC@UofSC) repository [21], an ongoing cross-sectional cohort study. Institutional Review Board approval was obtained, followed by written informed consent provided by all participants at enrolment.

### Neuroimaging acquisition and preprocessing

Participants underwent MRI scanning on a Siemens Trio 3T scanner with a 20-channel head coil. T1-weighted images were used for brain age estimation and were acquired using the following parameters: T1-weighted imaging (MP-RAGE) sequence with 1mm isotropic voxels, 256x256 matrix size, 9° flip angle, and 92-slice sequence with repetition time (TR)=2250ms, inversion time (TI)=925ms, and echo time (TE)=4.11ms. Fluid attenuated inversion recovery (FLAIR) scans were also acquired on the same scanner using the following parameters: TR = 5000 ms, TE = 387 ms, matrix = 256 x 256, FOV = 230 x 230 x 173 mm^2^, 1mm isotropic voxels, 160 sagittal slices.

WMHs were manually delineated on the FLAIR images in accordance with the STRIVE protocol (Standards for Reporting Vascular Changes on Neuroimaging) [17] by a trained individual (author SW) blinded to demographic information. The WMH load was calculated as the volume in mm^3^ (the total number of voxels) corresponding the WMH.

### Brain age estimation

Brain age estimation was performed based on T1-weighted images using the BrainAgeR analysis pipeline (github.com/james-cole/brainageR) [3, 19]. The T1-weighted images were segmented and normalized using SPM12's DARTEL toolbox [22]. Probabilistic tissue maps were visually inspected by a neurologist to ensure quality of the segmentation. Gray and white matter probabilistic tissues were entered into a machine-learning algorithm using a pretrained Gaussian regression model implemented in R-package Kernlab to estimate brain age.

The difference between estimated brain age and chronological age (brain age gap: BrainGAP) was determined by subtracting an individual’s chronological age from their estimated brain age. Therefore, the BrainGAP corresponded to premature or delayed brain age beyond chronological age [23]. Positive values suggest that the predicted brain age is older than the chronological age of the participant (i.e., premature brain aging) whereas negative values suggest that an individual’s brain age is younger than their chronological age (i.e., delayed brain aging).

### Behavioral testing

Participants were administered both standardized and informal measures of cognition and language on a laptop (MacBook Pro) or an iPad. Researchers administering the cognitive battery had C-level qualifications and obtained a cognitive unlock code in accordance with the NIH guidelines. Cognitive measures included the Montreal Cognitive Assessment (MoCA) [24], a cognitive screening assessment sensitive to mild cognitive impairment. Researchers completed training for administration and scoring of the MoCA. The total MoCA score is based on the following cognitive domains: attention and concentration, executive functions, memory, language, visuoconstructional skills and orientation. Total MoCA scores were calculated using the rules provided by the National Alzheimer’s Coordinating Center Uniform Data Set instruction manual for neuropsychological testing battery [25].

### Statistical analysis

As WMH volume increased exponentially with older age, we log-transformed the WMH volume data. This log-transformed variable is used in all future analyses. Pearson correlation coefficients were calculated between chronological age and estimated brain age, BrainGAP and WMH volume. Partial Pearson correlations were conducted between BrainGAP and WMH volume, accounting for chronological age. To investigate the relationship between these measures of cortical integrity and behavior, partial Pearson correlations were conducted between these measures and MoCA scores. All statistical analyses were conducted in the statistical software R (R Core Team, 2017), applied using R package NLME [26] and all figures were created using the GGPLOT2 package [27].

### Data availability

The data that support the findings of this study are available from the corresponding author upon reasonable request.

## RESULTS

**Figure 1:**
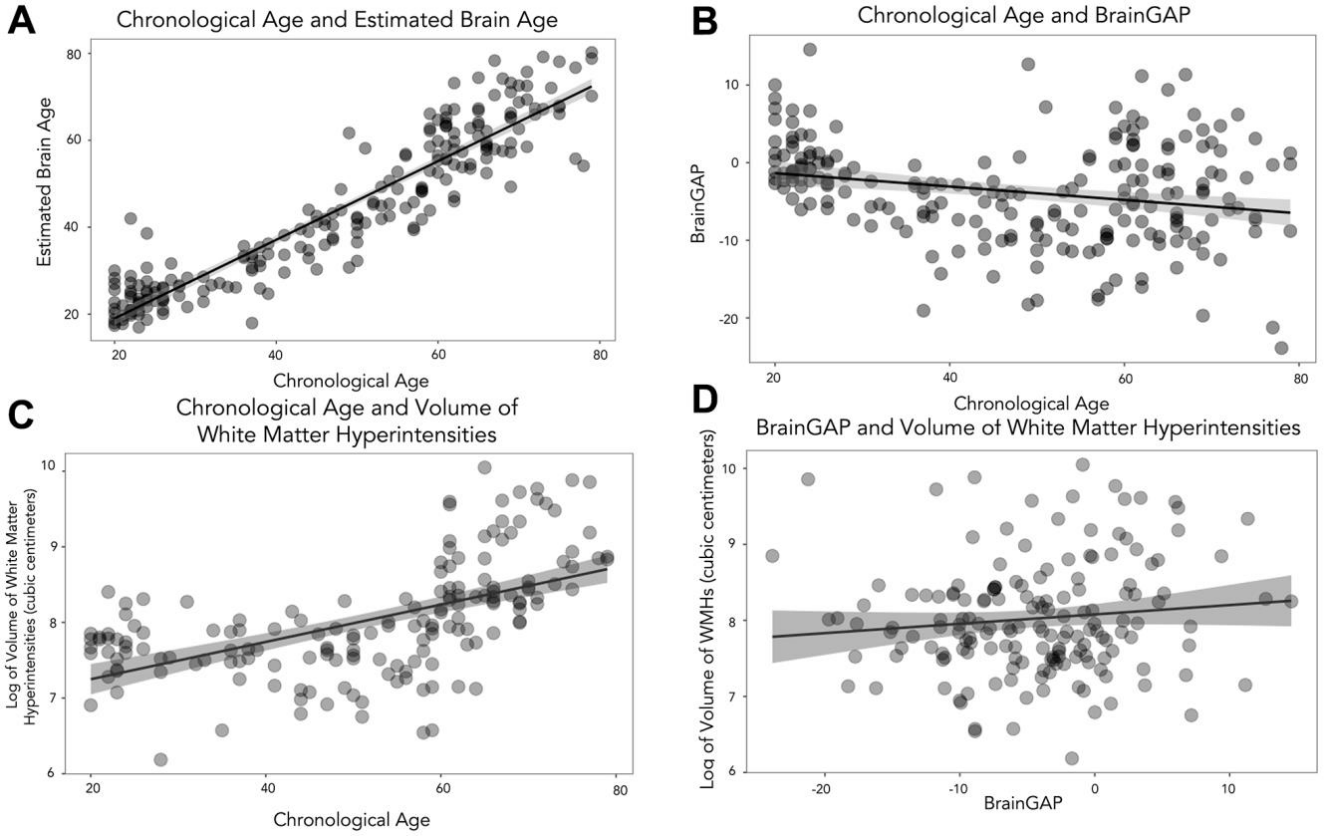
**Scatterplots to show the relationship between chronological age, brain age and volume of WMHs.** (**A**) shows chronological age and estimated brain age. (**B**) depicts chronological age and BrainGAP. (**C**) displays chronological age and volume of WMHs, and (**D**) shows BrainGAP and volume of WMHs.

Participants had an average chronological age of 47.35 years (*SD*=18.33, range=20-79). Similarly, they had an average brain age of 43.76 years (*SD*=17.80, range=16.95-80.22). There was a significant positive correlation between chronological age and estimated brain age (Pearson*: r*(163)=0.932, *p*<0.001), see Figure 1A.

The average BrainGAP was -3.72 years (*SD*=6.48, range=-23.90-14.57). There was a significant negative correlation between chronological age and BrainGAP (Pearson: *r*(163)=-0.244, *p*<0.001), see Figure 1B.

Participants had an average of 4.17 cubic centimeters of WMH (*SD*=4.02, range=0.49-23.12). There was a significant positive correlation between chronological age and volume of log-transformed WMH (Pearson*: r*(163)=0.560, *p*<0.001), see Figure 1C.

**Figure 2:**
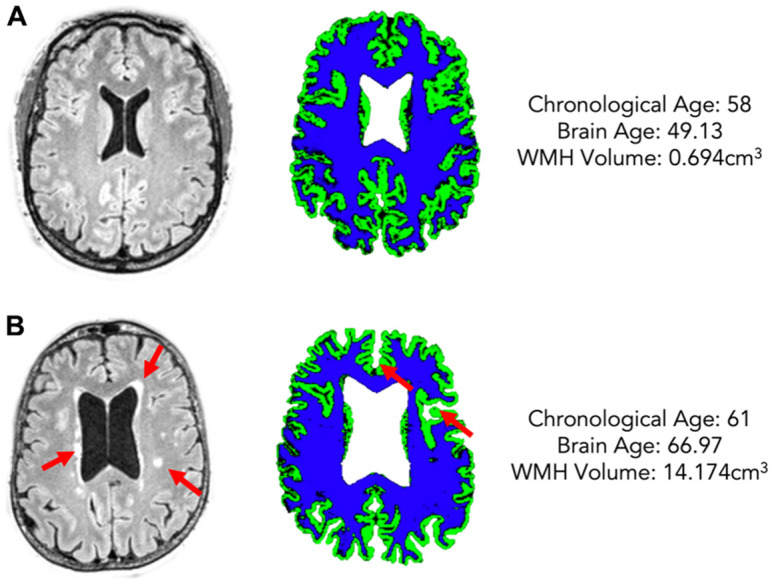
Two example participants of a similar chronological age (**A**: 58, **B**: 61) but different estimated brain age (**A**: 49.13, **B**: 66.97) and different WMH volume (**A**: 0.694cm^3^, **B**: 14.174cm^3^). The left column shows FLAIR scans with WMHs highlighted by red arrows, the right shows grey matter (green) and white matter (blue) maps for each participant.

Due to the significant relationship between chronological age and BrainGAP (likely related to floor and ceiling effects as the BrainAgeR model was initially trained on participants older than 20 and younger than 80) and chronological age and volume of WMHs, we conducted partial Pearson correlations (accounting for chronological age) between BrainGAP and volume of (log-transformed) WMHs which revealed a significant relationship (Pearson: *r*(163)=0.233, *p*=0.003), see Figure 1D. See Figure 2 for example participants. Based on the partial correlation between BrainGAP and WMH (controlling for chronological age) each additional year in brain age corresponded to additional 1.1mm^3^ in WMH load.

Partial correlations, accounting for chronological age, revealed that BrainGAP and WMH volume were significantly associated with different aspects of cognition. Specifically, BrainGAP was significantly associated with the MoCA words generated sub score (*r*(163) = -0.186, *p* = 0.008), where premature brain aging is associated with worse scores, but there was no significant relationship with MoCA total score (*p* = 0.357) or any other MoCA sub score (*p* > 0.05). Conversely, WMH volume was significant associated with MoCA total score (*r*(163) = -0.199, *p* = 0.005) and the MoCA attention and concentration index score (*r*(163) = -0.221, *p* = 0.002), where increased WMH volume was associated with poorer scores. No other significant relationship with MoCA sub scores were found.

## DISCUSSION

Brain age is a measure of brain integrity that captures age-related atrophy in the brain independent from chronological age. Conversely, WMHs are a measure of white damage due to small vessel disease. In this study, we investigated the independent association between quantifiable WMHs and brain age in an original aging cohort, and the association between these markers and cognition. Our results support previous work suggesting there is a relationship between chronological age and WMH volume, and chronological age and estimated brain age. Our results also demonstrate that there is a significant association between WMHs and premature brain age when accounting for chronological age. BrainGAP and WMH burden were associated with different aspects of cognition, where WMH burden was associated with reduced overall cognition (MoCA total score) and premature brain aging was associated with poorer language fluency.

This original cohort study is the first to use quantifiable measures of WMHs alongside machine learning-derived brain atrophy measures (using the BrainAgeR pipeline) and demonstrated a strong association between WMHs and premature brain age. These results indicate a relationship between WMHs and premature brain aging, suggesting that there is either a direct link between small vessel disease and premature brain aging, or that processes shared a common causal link. Although significant, the correlation coefficient is relatively weak which may support the theory of a shared common causal link which may influence premature brain aging and small vessel disease at slightly different rates or through different mechanisms. Importantly, these results underscore the importance of overlapping factors related to brain health. WMHs are common in older age and are related to cardiovascular risk factors such as body mass index (BMI), hypertension or diabetes [28]. Albeit previously considered a finding of uncertain significance, more recent evidence suggests that WMH are related to lowered cognitive performance [29–31]. Taken together, WMHs can be considered a marker of brain health [32, 33] and, as these results indicate, a factor associated with premature brain aging. Similarly, premature brain aging has also been previously associated with higher cardiovascular burden [15] and declines in cognitive performance [34, 35].

It is unclear if premature brain aging shares the same pathophysiology compared with WMHs and is therefore an irreversible process. Nonetheless, premature brain aging is also increasingly recognized as a potent marker of lowered cognitive skills [34, 36, 37]. Its association with WMHs demonstrated here suggests that small vessel disease can be part of the process related to premature aging and underscores the importance of multiple converging factors supporting brain health and progressive declines in cognitive ability [29–31]. This is further supported by our results demonstrating that WMH burden and premature brain aging may differentially affect cognition and therefore provides a potential explanation for why some individuals experience different rates of cognitive decline in different aspects of cognition (i.e., memory, language etc.). The relationship between WMH load and general cognition (MoCA total score) is supported by previous literature [29–31], however the relationship found here between premature brain aging and language fluency is less well established. The differences in associated behavioral change may be related to the brain regions affected, as WMHs typically affect subcortical structures whilst premature brain aging is associated with a reduction in cortical integrity. It may be that brain regions such as the temporal lobe are particularly affected with premature brain aging and may explain why we found a relationship with BrainGAP and language fluency, however future studies should be conducted to investigate this further. Premature brain aging has also been found in many common brain disorders, including schizophrenia, multiple sclerosis, and dementia [38]. Similarly, small vessel disease and, in particular, white matter hyperintensity severity has also been associated with mild cognitive impairment and dementia [39, 40] Therefore, future research could investigate the interaction between brain age and white matter hyperintensity load in different disorders.

### Limitations

The main limitation of this study is the relatively small sample size (N=166) compared to other datasets. However, the current cohort has associated behavioral data (MoCA scores) which is not available for the majority of the larger open access databases. This allows investigation of the relationship between markers of cortical integrity and different aspects of cognition which is not possible using larger datasets with only MRI-based information. However, future studies could investigate the relationship between WMH volume and brain age using the BrainAgeR analysis pipeline on larger datasets such as UK Biobank.

## CONCLUSIONS

This study corroborated previous work which has found a relationship between chronological age and WMH volume, and chronological age and estimated brain age. Our results also demonstrate that a higher WMH load (a marker of small vessel disease) is associated with premature brain aging (a measure of brain integrity which captures age-related atrophy independent of chronological age), and that both are associated with different aspects of cognitive decline. Future research could investigate the interaction of premature brain aging and WMH load on behavior in different cognitive disorders.
